# The Biodegradation of Soil Organic Matter in Soil-Dwelling Humivorous Fauna

**DOI:** 10.3389/fbioe.2021.808075

**Published:** 2022-01-10

**Authors:** Xuliang Lou, Jianming Zhao, Xiangyang Lou, Xiejiang Xia, Yilu Feng, Hongjie Li

**Affiliations:** ^1^ Zhuji Real Estate Management Service Center, Shaoxing, China; ^2^ State Key Laboratory for Managing Biotic and Chemical Threats to the Quality and Safety of Agro-products, Key Laboratory of Biotechnology in Plant Protection of Ministry of Agriculture and Zhejiang Province, Institute of Plant Virology, Ningbo University, Ningbo, China

**Keywords:** soil organic matter, biodegradation, humivorous, biotechnology, enzyme

## Abstract

Soil organic matter contains more carbon than global vegetation and the atmosphere combined. Gaining access to this source of organic carbon is challenging and requires at least partial removal of polyphenolic and/or soil mineral protections, followed by subsequent enzymatic or chemical cleavage of diverse plant polysaccharides. Soil-feeding animals make significant contributions to the recycling of terrestrial organic matter. Some humivorous earthworms, beetles, and termites, among others, have evolved the ability to mineralize recalcitrant soil organic matter, thereby leading to their tremendous ecological success in the (sub)tropical areas. This ability largely relies on their symbiotic associations with a diverse community of gut microbes. Recent integrative omics studies, including genomics, metagenomics, and proteomics, provide deeper insights into the functions of gut symbionts. In reviewing this literature, we emphasized that understanding how these soil-feeding fauna catabolize soil organic substrates not only reveals the key microbes in the intestinal processes but also uncovers the potential novel enzymes with considerable biotechnological interests.

## Introduction

Soil organic matter (SOM) is massive and representative of a major organic carbon pool on the planet, which is considered as an essential agent in maintaining ecosystem productivity and sustainability through its physical, chemical, and biological properties. More specifically, soil organic matter not only retains nutrients that improve plant growth but also contributes soil physicochemical property enhancements such as infiltration, water-holding capacity, and aggregates (Lehmann and Kleber, 2015). To date, researchers estimate SOM approximately makes up less than 5% of the global dry weight soil (Stevenson, 1972; Liang et al., 2017). Soils also contribute an important source of aquatic and atmospheric carbon; moreover, diverse living organisms within the soils are considered as the most driving force of carbon cycling in biogeochemical processes. Collectively, organic matter in the soil represents the most abundant source of organic carbon and has unparalleled ecological and economic impacts on the Earth (Bolan et al., 2011).

The formation and turnover of soil organic matter is a continuum of progressively decomposing processes. Biological, physical, and chemical transformation processes convert dead plant material into organic products that form intimate associations with soil minerals (Lehmann and Kleber, 2015). The fragments of plants are often first broken up into small pieces at the beginning of decomposition by soil fauna. The plant residues are further degraded by subsequent exo-enzymes derived from surrounding microorganisms, where they are broken down to a relatively small size. The generated organic compounds at various stages of decay not only represent energy-rich spots in the soils but also relatively recalcitrant components. For instance, polyphenols in soils exist either in a dissolved form that moves freely in the soil solution, in a sorbed form that reversibly binds to the soil particle or proteins, or in a polymerized form that consists of humic substances (Min et al., 2015) Among them, lignin is one of the most recalcitrant carbon compounds and can bind with proteins, thereby immobilizing nitrogen (Gentile et al., 2011). Increasing evidence shows that soil-dwelling fauna and their gut microbial symbionts have the ability to decompose these persistent materials even more quickly than previously recognized (Coleman and Wall, 2015). In this review, we provide an overview of the recent omics-based research, including soil-dwelling fauna and their associated gut bacterial genomic and metagenomic studies, which have led to a deeper understanding of soil organic matter degradation processes and uncovered the presence of only recently recognized microbial symbionts and relevant degradative enzymes.

## The Chemical Complexity and Recalcitrance of Soil Organic Matter

**FIGURE 1:**
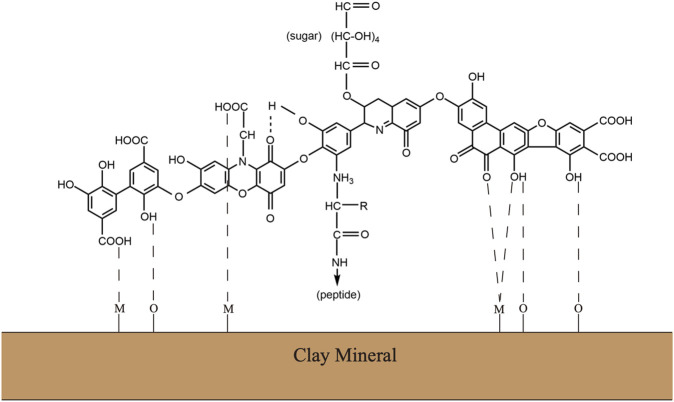
Traditional view of the chemical complex of soil organic matters or humus substance. Modified from Wagner and Wolf (1998).

Soil organic matter is heterogeneous complexes with a variety of chemical components. Although the definite chemical structures have remained contentious, it is generally accepted that humic substances consist of polyphenols, peptides, lipids, and polysaccharides (Figure 1) (Gerke, 2018). This supramolecular network formed by complex carbohydrates and aromatic polymers provides the SOM complexes with sufficient stability, but it also makes the SOM a major barrier to gain access to the stored hydrolysable aliphatic components (Horwath and Paul, 2015). The substantial ether– and carbon–carbon interunit linkages between aromatic units possess an inherent chemical recalcitrance. At the same time, the SOM often has chemical interaction with inorganic soil colloids, including mineral or clay particles, to form dense aggregates, which further provides the physical protections against decomposition (Oades and Waters, 1991). Specifically, owing to the stimulation of microbial activity and microbe-derived carbon, plant residue starts to form aggregates when it enters the soil. Along with the decomposition processing, plant residues or other particulate organic matter gradually encrusted with clay particles and microbial byproducts to form the core of stable microaggregates. Consequently, the mineral crusts interacting with microbial byproducts managed to form recalcitrant organo–mineral complexes (Six et al., 2004).

The stability of soil organic matter including peptides, amino acids, and polysaccharides is strongly related to the presence of humic substances, which is largely owing to the polymerization of aromatic units during the humification (Shan et al., 2010). Modern analytical approaches for characterization of biomolecules in microbial cells and soils now suggest a direct and rapid contribution of microbial cell walls to soil organic matter protections when associated with model polyphenolic components. The emergent concept of soil organic matter as a continuum spanning the full range from intact plant material to highly oxidized carbon in carboxylic acids represents the more common view among the public (Lehmann and Kleber, 2015).

## Biodegradation Mechanism of Soil Organic Matter

Soil organic matter degradation mechanisms in natural systems have remained less known since their structural complexes and therefore a suite of ligninolytic enzymes are likely engaged in the degradation of humic substances, such as lignin peroxidase (LiP), manganese peroxidase (MnP), versatile peroxidase (VP), and laccase (Abdel-Hamid et al., 2013).

Among organisms, actinobacteria and fungi are most well known to be capable of degrading humic substances. The fungi involved in humic acid degradation are usually known as white-rot fungi capable of lignin degradation (Dashtban et al., 2010; Datta et al., 2017). Extracellular enzymes including laccase and ligninolytic peroxidase are involved in the cleavage of aromatic rings; among them, manganese peroxidase is the most investigated (Nousiainen et al., 2014). Also, protease, lipase, and various carbohydrases may be involved in the degradation of aliphatic structural components (peptides, lipids, polysaccharides, etc.) (Holtof et al., 2019). Enzymatic degradation of protein from humic acids has been demonstrated. Meanwhile, the release of amino acids from humic substances by chemical autoxidation has also been observed (Kappler and Brune, 1999).

## The Mechanism and Process of Soil-Dwelling Macrofauna Breaking Down the Soil Organic Matter

Some soil fauna feed on soil organic matter, exclusively relying on soil organic matter in an advanced stage of humification (Briones, 2014). In fact, Donovan et al. (2001) defined four feeding groups of soil fanuas based on the humification stages of their gut content: 1) feeding on wood, litter, and grass; 2) feeding on very decayed wood and/or high organic content soil; and 3) feeding on only organic soil (so-called true soil feeders) (Donovan et al., 2001). The mineralization of SOM components throughout the guts of soil-feeding fauna has a significant impact on carbon-cycling globally. Indeed, several soil-dwelling fauna evolved the capacity to efficiently utilize the stored organic carbon within the soil organic matter (Jiang et al., 2018). Given the independent evolution of different soil-dwelling fauna, diverse bioprocessing mechanisms of the soil organic matter–based diet across these organisms have been established. The major innovation in soil fauna is a variety of microbes and their relevant enzymes engaged in these biodegradation processes, which either hydrolyze residual polysaccharides or degrade polyphenolic components of soil organic matter.

In the natural ecosystem, there is a diverse population of soil-dwelling fauna; among them, most research concentrates on earthworms, beetles, and termites (Swift et al., 1979; Li et al., 2021). Organic matter transformation is directly affected by soil macrofauna through the incorporation and redistribution of various materials and indirectly by making use of the microbial community with both constructive and destructive means (Wolters, 2000; Lavelle et al., 2001; Liu et al., 2019). More current research studies concentrate on the representative soil organisms including earthworms, beetles, and termites, which ingest a mixture of organic matter, soil components, and microorganisms adhering to mineral particles (Mcquillan and Webb, 1994; Lavelle et al., 1997; Brauman et al., 2000). Highly compartmentalized gut structure, extremely alkaline gut microenvironment, hydrolytic enzymes, and specialized microbiota in the gut of soil-dwelling fauna are the key points in the digestion of organic matter.

## The Conversion of Soil Organic Matter in Earthworms

**FIGURE 2:**
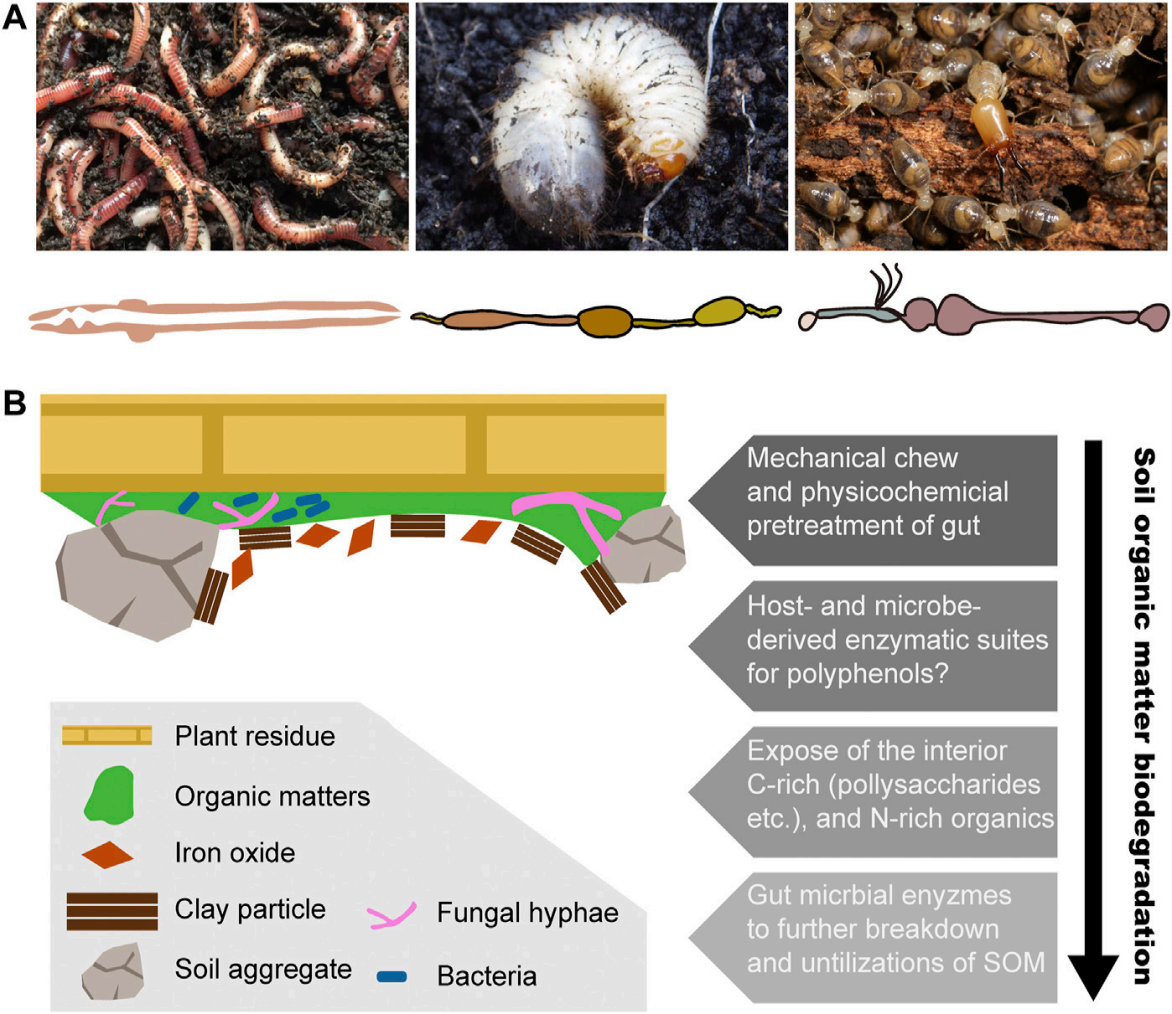
Biodegradation process of soil organic matter in three representative humivorous fauna. **(A)** Humivorous earthworm, beetle larva, and higher termites, as well as their gut morphology. Termite photo image courtesy of Jan Šobotnĺk. **(B)** Structural complex and heterogeneity of soil organic matter and the hypothetical biodegradation mechanism among soil-feeding macrofauna.

Earthworms live in diverse types of soil, ranging from the top of soil in the surface litter, rotting logs, and the axils of tree branches, to the moist soil surrounding natural freshwater bodies (Reynolds, 1994). Earthworms contribute huge ecological impacts by modifying the soil structure. For example, the tropical earthworm *Reginaldia omodeoi* can take up to 30 times its own biomass of soil per day through its simple and tubular gut (Figure 2A) (Blouin et al., 2013). In temperate ecosystems, earthworms also ingest large amounts of material, with approximately 2–15% of organic matter inputs (Whalen and Parmelee, 2000). Earthworms live in the soil and ingest a mixture of soil and organic matter and finally excrete organo–mineral feces. Some species are dwellers and transformers of litter, living in organic soil horizons in or near the surface litter, with a diet of coarse particulate organic matter. This species takes large amounts of undecomposed litter and excretes holorganic fecal pellets (Dominguez and Edwards, 2010). Consequently, incorporation of organic matter into soil and the formation of macroaggregates are finished through burrowing, consumption, and egestion activities of earthworms (Guggenberger et al., 1996; Blanchart et al., 1997). After digestion, nitrogen is also reused by plants so that in the presence of earthworms, nitrogen mineralization increases either directly through the release of nitrogen by their metabolic products and dead tissues or indirectly through changes in soil physical properties and fragmentation of organic material and through interactions with other soil organisms (Lee, 1985; Bityutskii et al., 2002).

Research studies about degradation of soil organic matter by earthworms are currently focused on the degradation and transformation of plant-derived materials, such as cellulose, lignin, and other components of plant litter (Angst et al., 2021). Early feeding experiments on earthworms by using ^14^C-labeled lignin substrates indicate that the effect of earthworms on the degradation of cellulose and lignin has two distinct aspects: promotion of initial biodegradation and inhibitory effect of lateral biodegradation (Scheu, 1993). In holocellulose mineralization, earthworm processing causes a two-phase alteration: mineralization rates were initially increased for 6–15 weeks but decreased later in the experiment. Overall holocellulose mineralization in the soil of the 6- and 13-year-old fallows was increased by factors of 1.5 and 1.4 due to earthworm processing, respectively, whereas in wheatfield and beechwood soil, the effects are only slight. In the case of wheatfield soil, the earthworm processing causes a two-phase alteration in the context of the rate of lignin mineralization: mineralization rates were increased for about 10 weeks but decreased afterward in the experiment. Moreover, these earthworms have much a higher degradation capacity on cellulose than on lignin (Scheu, 1993).

In earthworms, the gut community is dominated by the Proteobacteria, Acidobacteria, Actinobacteria, Firmicutes and Verrucomicrobia taxa within three genera of earthworms, *Aporrectodea*, *Allolobophora*, and *Lumbricus* (Sapkota et al., 2020). Several microbiome analysis results of different earthworms indicate that Proteobacteria is likely the most abundant in the gut microbiota (Knapp et al., 2009; Liu et al., 2011; Liu et al., 2018), which is consistent with early reports that Proteobacteria might be involved in the fermentation, digestion, and absorption of food for the earthworm host (Flint et al., 2012).

## Selective Digestion of Polysaccharides of SOM in Humivorous Larva of Beetles

Among beetles, most larvae feed on fresh or decomposing vegetable materials (Wolters, 2000). In the case of the Scarabaeidae beetle *Pachnoda ephippiata*, the larvae are considered almost entirely herbivorous or saprophagous (Crowson, 2013). The intestinal tract of Scarabaeid beetle larvae is mainly composed of two enlarged components, the long tubular midgut and a paunch hindgut, but also a poorly developed foregut (Figure 2A) (Cazemier, 1999). It has been observed that in saprophagous beetle larvae, the gut contains not only a large amount of humic material and plant residues but also fungal hyphae and other microbes (Bauchop and Clarke, 1977; Crowson, 2013). In Scarabaeidae families, similar to many soil-feeders, alkaline pH (>10) is always found in the midgut. The recalcitrant chitin and peptidoglycan, also the major structural polymers in the soil organic matter, are co-polymerized with polyphenols and thereby more likely to be against the soil microbial degradation. Early feeding experiments reveal that *P. ephippiata* larvae enable the selective digestion of those two polysaccharides over the protections from the polyphenols (Li and Brune, 2005).

The bacterial community structure study of the *P. ephippiata* larvae gut indicates the presence of dense and diverse microbiota, which is considerably different to the surrounding soils (Egert et al., 2003; Lemke et al., 2003). One of the dominant bacterial species isolated from the hindgut of the larvae, *Promicromonospora pachnoda*e, is capable of reducing iron and degrading (hemi)cellulose (probably simultaneously), which indicates that dissimilatory iron reduction is involved in the degradation of organic matter in the intestinal tract. Also, other substantial cellulolytic bacteria, hemicellulolytic bacteria, and methanogenic archaea have been found in the intestinal tract (Bayon and Mathelin, 1980).

In some dung beetles, microbiome research studies of *Onthophagus* beetles reveal that Enterobacter and Serratia are the dominant genera in the adults, while Dysgonomonas and Parabacteroides dominate in larval and pupal stages (Suárez-Moo et al., 2020). Nevertheless, the genus *Dysgonomonas* is more abundant in the larval stage of *E. intermedius* and *E. triangulatus* (Shukla et al., 2016) and the gut microbiota of two *Pachysoma MacLeay* desert dung beetle species (Franzini et al., 2016).

## Mobilization and Transformation of Nitrogenous Components Within SOM in Humivorous Higher Termites

Termites consist of seven families and are phylogenetically classified into lower termites with six families and higher termites with just one family (Noirot, 1992). For the wood-feeding “lower” termites, cellulolytic protozoa and bacteria attribute the plant biomass digestions. Evolutionarily derived “higher” termites, which are completely lacking in protozoa, have an extensive diet diversity ranging from wood, grass, bark, lichen, and decayed litter to organic soil (Wood, 1978). Among them, soil-feeding species are found in three subfamilies of higher termites and constitute approximately 67% of all genera (Brauman et al., 2000). Soil-feeding termites have been considered as important contributors to biogeochemical cycles, especially in carbon, methane, and nitrogen (Sugimoto et al., 2000; Ji and Brune, 2006). In the tropical savanna, termites have been estimated to be directly responsible for up to 20% of total carbon mineralization (Lavelle et al., 1997).

In soil-feeding termites, the hindgut is highly compartmentalized and longer, which is classified in five sections, from P1 to P5 (Figure 2A) (Brune and Kühl, 1996). It is observed that in comparison with the generally tubular compartments of wood feeders, humivorous higher termites are characterized by dilated P1 compartments, which is characterized by an increase in the length and volume, so that it allows a sequential transit of long duration. Notably, the pH sharply increases in the mixed segment and results in the alkalinity in the anterior hindgut of soil feeders being the highest values that have been reported for biological systems (Wang, 2018).

Early studies have already estimated the strong mineralization of carbon and nitrogen in the gut of soil-feeding termites, even though the overall information on humivorous termites is still limited. Ji and Brune (2005) found that soil-feeding termites, *Cubitermes orthognathus*, enable the efficient mobilization and digestion of the peptidic components within the soil organic matter by a combination of proteolytic activities and extreme alkalinity in their intestinal tract (Ji and Brune, 2005). By using pyrolysis-GC-MS, Griffiths et al. (2013) further confirms that in comparison to the wood-feeding termites, the soil-feeder *Cubitermes* termites efficiently digested peptides and other nitrogenous residues such as chitin and peptidoglycan of soil organic carbon, rather than polyphenols (Griffiths et al., 2013). Interestingly, nitrogenous components are derived from microbial biomass, which are generally protected from degradation by covalent linkage to polyphenols and an intimate association with clay minerals. The ability to mobilize such recalcitrant humus constituents is accompanied by an even more pronounced elongation and extreme alkalization (to >pH 12) of the anterior hindgut, which remains a mystery.

Diverse and unique microbial populations exist in the hindgut of soil-feeding termites. Termites largely depend on these complex microbial communities to digest and utilize soil organic matter, including highly recalcitrant lignocellulose and other organic matters in advanced stages of humification (Ohkuma and Brune, 2011). It has been demonstrated that the relative increase in alkali-active proteases in the P1 section and ammonia accumulates to high concentrations in the posterior hindgut. The magnified abundance of these alkali-adapted Firmicutes belongs to clostridia in their hindguts may satisfy the metabolic requirement (Mikaelyan et al., 2015). However, the concrete roles played by intestinal microbiota in the digestive process are still unclear.

To date, there are numerous gut microbiome studies across feeding groups of termites. The overall pattern indicates a prevalence of Fibrobacteres and Spirochaetes bacteria in the wood feeders, whereas humus feeders, soil feeders, and fungus feeders shared similarities in community structure, with large proportions of Firmicutes, Bacteroidetes, and Proteobacteria. Furthermore, the soil feeders also harbored a larger proportion of actinobacteria (Schloss et al., 2006; Dietrich et al., 2014; Su et al., 2016; Bucek et al., 2019; Hu et al., 2019). The latest work on the large-scale metagenomic analysis of 145 termite species revealed the correlation between host phylogeny and the functionalities of their microbiota (Arora et al., 2021).

## Microbiome of Soil-Dwelling Humivorous Fauna and Bioenergy Applications

As one of the largest carbon pools, soil organic matter represents a complex and recalcitrant carbon that has an inherent resistance to decomposition, which is largely owing to the protection provided by soil minerals and a variety of aromatic biopolymers (Kleber, 2010). The ability of decomposer soil fauna to access the stored carbon of soil organic matter at an incredibly efficient level has fascinated biologists for more than a century. In parallel to the current industrial saccharification, the breakdown of complex polysaccharides into monosaccharides employs strategies involving a combination of chemical pretreatment and enzymatic hydrolysis to obtain simple sugar for subsequent fermentation (Hafid et al., 2017). The depolymerization processing is still not economically viable and is even challenging.

Notably, soil-dwelling fauna is widespread on Earth, for example, soil-feeding termites inhabit approximately 75% of the terrestrial soil surface and consume wood and litter in different stages of decay and humification (Noirot, 1992; Li et al., 2017). The microbiome of soil-dwelling humivorous fauna represents a particularly vast and promising source of novel cellulolytic enzymes, or enzyme cocktails, for industrial cellulosic biofuel production. Yet, we have only begun to understand the ecologic impacts. Work on the core and functional bacterial lineages and their related microbial enzymes and genomic investigations have led to discoveries of novel and diverse microbe-derived enzymes. To further explore these biological systems, it is essential to proceed beyond a full understanding of the chemistry of the nature of all organic matter in soil. An integrative analysis of chemically tracking the fate of soil organic matter throughout soil-dwelling humivorous fauna is urgently necessary.
